# Molecular Characterization of *Gyrovirus galga* 1 in Domestic Dogs in the North of Vietnam Indicates the Presence of Recombination Events

**DOI:** 10.3390/ijms27031384

**Published:** 2026-01-30

**Authors:** Giang Huong Thi Tran, Amonpun Rattanasrisomporn, Dao Anh Tran Bui, Hieu Van Dong, Jatuporn Rattanasrisomporn

**Affiliations:** 1 Faculty of Veterinary Medicine, Vietnam National University of Agricuture, Hanoi 131000, Vietnam; tthgiang@vnua.edu.vn (G.H.T.T.); btadao@vnua.edu.vn (D.A.T.B.); dvhieuvet@vnua.edu.vn (H.V.D.); 2 Interdisciplinary of Genetic Engineering and Bioinformatics, Graduate School, Kasetsart University, Bangkok 10900, Thailand; fgraapr@ku.ac.th; 3 Department of Companion Animal Clinical Sciences, Faculty of Veterinary Medicine, Kasetsart University, Bangkok 10900, Thailand

**Keywords:** *Gyrovirus galga* 1, Vietnam, PCR, genetic characterization, recombination

## Abstract

A total of 168 fecal samples were obtained from both diarrheic dogs exhibiting clinical symptoms and clinically healthy individuals across four provinces/cities in Northern Vietnam. Based on a polymerase chain reaction method, the Gyrovirus gala 1 (GyVg1) genome was found in 18 (10.71%) of 168 samples. Six representative GyVg1 genomes identified in Vietnam were each 2375 nucleotides long. They shared nucleotide identities of 94.98–98.62%. Whole genome phylogenetic analysis showed that the six obtained GyVg1 strains were grouped into two distinct lineages: GyVg1 I (2/6 strains) and GyVg1 II (4/6 strains). Vietnamese GyVg1 strains were genetically related to Chinese strains. Multiple residue replacements were identified in VP (VP1–VP3) proteins of the Vietnamese GyVg1 strains. A recombination event led to the emergence of the GyVg1/Dog/VNUA-06 recombinant strain. Overall, these observations indicate the presence of GyVg1 viruses in domestic dogs in the North of Vietnam.

## 1. Introduction

*Gyrovirus galga* 1 (GyVg1), formerly known as avian gyrovirus 2, belongs to the Gyrovirus genus of the *Anelloviridae* family. GyVg1 possesses a circular single-stranded DNA genome measuring about 2375 to 2376 nucleotides in length [1]. In 2011, human gyrovirus (HGyV) was detected in skin swabs collected from healthy humans. The HGyV virus was found to share 96% genomic sequence similarity with GyVg1, which was initially discovered in diseased chickens from Brazil [2].

The *Anelloviridae* family comprises 31 taxonomically established genera; notably, the Gyrovirus genus is known to infect various avian hosts, whereas the remaining 30 genera predominantly infect humans and other mammalian species [3,4]. GyVg1 was first discovered in chickens, which are generally regarded as its original hosts. Nevertheless, the genome of HgyV/GyVg1 was also detected in blood samples from organ-transplant patients and human immunodeficiency virus-positive individuals in Italy, as well as from healthy blood donors in France [5,6]. In addition, previous reports indicated that GyVg1 has been found in various host species, including dogs, cats, and ferrets, in addition to several mammalian and avian species in zoological environments [2,7,8,9].

The GyVg1 genome is approximately 2.37 to 2.38 kb, including three open reading frames (ORFs) with overlapping regions along with an untranslated region (UTR) [1]. Among these, ORF1 encodes the VP2-scaffolding protein consisting of 225 amino acids (aa). Additionally, the conserved motifs CX5R and WX7HX3CXCX5H are also commonly found in the VP2 protein of other Gyroviruses. The ORF2 region codes for the VP3 protein, a 111 aa protein known as Apotin. This protein has been considered to induce apoptosis in hematopoietic cells and T lymphocytes in chickens, thereby resulting in promoting apoptotic death in tumor cells and anemia symptoms in affected chickens [10]. ORF3 codes for the viral capsid VP1 protein composing 453 aa. VP1 presents the structural protein of Gyroviruses. In addition, the presence of VP2 during expression is necessary for the generation of neutralizing antibody responses [11].

More recently, the evolution of city lifestyles has increased the importance of companion animals, notably dogs and cats, in daily human activities. The close and regular interactions between companion animals and humans have been considered to facilitate the transmission of emerging viruses, positioning pets as potential carriers or intermediate hosts. Multiple Gyrovirus strains, consisting of GyVg1, were reported in domestic cats in Northeastern China in 2019 [12]. Subsequently, GyVg1 was detected in serum samples collected from domestic dogs in additional parts of China in 2022 [13]. Ongoing studies of GyVg1 have demonstrated an increasing trend in the detection frequency of this virus over time [14].

In Vietnam, GyVg1 has been reported [15]; however, the information is still limited. The epidemiological characteristics of GyVg1 in chickens and other potential hosts warrant further clarification. Thus far, GyVg1 has been documented in several regions worldwide [14,16,17,18]. Accordingly, further efforts are necessary to detect GyVg1 in companion animals, especially dogs, and to evaluate the genetic characteristics of the virus in order to clarify the current of GyVg1 circulation in Vietnam. Herein, the present study was carried out to detect and analyze the genome sequence of GyVg1 from domestic dogs to uncover insights into the genetic diversity and mutational characteristics of GyVg1 viruses.

## 2. Results

### 2.1. GyVg1 Genome Detection by PCR

In this study, samples were collected from domestic dogs, including both diarrheic dogs and clinically healthy individuals. PCR detection identified the GyVg1 genome in 18 of 168 (10.71%) samples. Specifically, the GyVg1 positive rates were 9.75% (8/82) in HN, 11.53% (3/26) in HP, 11.11% (5/45) in BN, and 13.33% (2/15) in HY (Table 1). No significant difference in the GyVg1 prevalence was found among the four provinces/cities (*p* > 0.05). Notably, GgVg1 genomes were detected in both diarrheic and asymptomatic dogs in the present study.

### 2.2. Genetic Analysis of the Full-Length Genome of GyVg1 Viruses from Vietnamese Domestic Dogs

Regarding molecular characterization, six positive samples with GyVg1 obtained from domestic dogs across multiple geographic locations (HN, HP, BN, and HY) were subjected to sequencing. The full-length genome sequences of current strains were designated as GyVg1/DOG/VNUA-01 (PX486819), GyVg1/DOG/VNUA-02 (PX1486820), GyVg1/DOG/VNUA-03 (PX1486821), GyVg1/DOG/VNUA-04 (PX1486822), GyVg1/DOG/VNUA-05 (PX1486823), and GyVg1/DOG/VNUA-06 (PX1486824), with short names following VNUA-01, VNUA-02, VNUA-03, VNUA-04, VNUA-05, and VNUA-06, respectively (Supplemental Table S1).

The whole genome sequence alignment and comparison (2375 nt) were performed for six current GyVg1 strains against reference sequences available in GenBank. Comparative analysis revealed that the six GyVg1 strains identified in this study shared nucleotide identities of 94.98–98.62%. Strains VNUA-02 and VNUA-05 exhibited the greatest similarity (98.62%), while strains VNUA-01 and VNUA-05 showed the lowest level of identity (94.98%) (Table 2). Furthermore, comparative analysis between the six current Vietnamese GyVg1 genomes and reference sequences available in the GenBank indicated that strains VNUA-02 and VNUA-05 shared 97.64–97.89% with DOG02 (Accession no. OR921200) and DOG04 (Accession no. OR921202), belonging to genotype cluster I. Conversely, strains VNUA-01, VNUA-03, VNUA-04, and VNUA-06 exhibited nucleotide identities ranging from 96.60% to 98.32% with DOG/AGV2-GXHG-32/2019 (Accession no. OK245349) and DOG/AGV2-GXBS-26/2019 (Accession no. OK245348), grouping them within genotype cluster II (Table 2). Moreover, the current GyVg1 strains shared 93.63–97.29% nucleotide similarity when compared with the GyVg1 strains from broiler chickens in Vietnam (Supplemental Table S2).

### 2.3. Phylogenetic Analysis of the Full-Length Genome of the GyVg1 Viruses from Vietnamese Domestic Dogs

**Figure 1:**
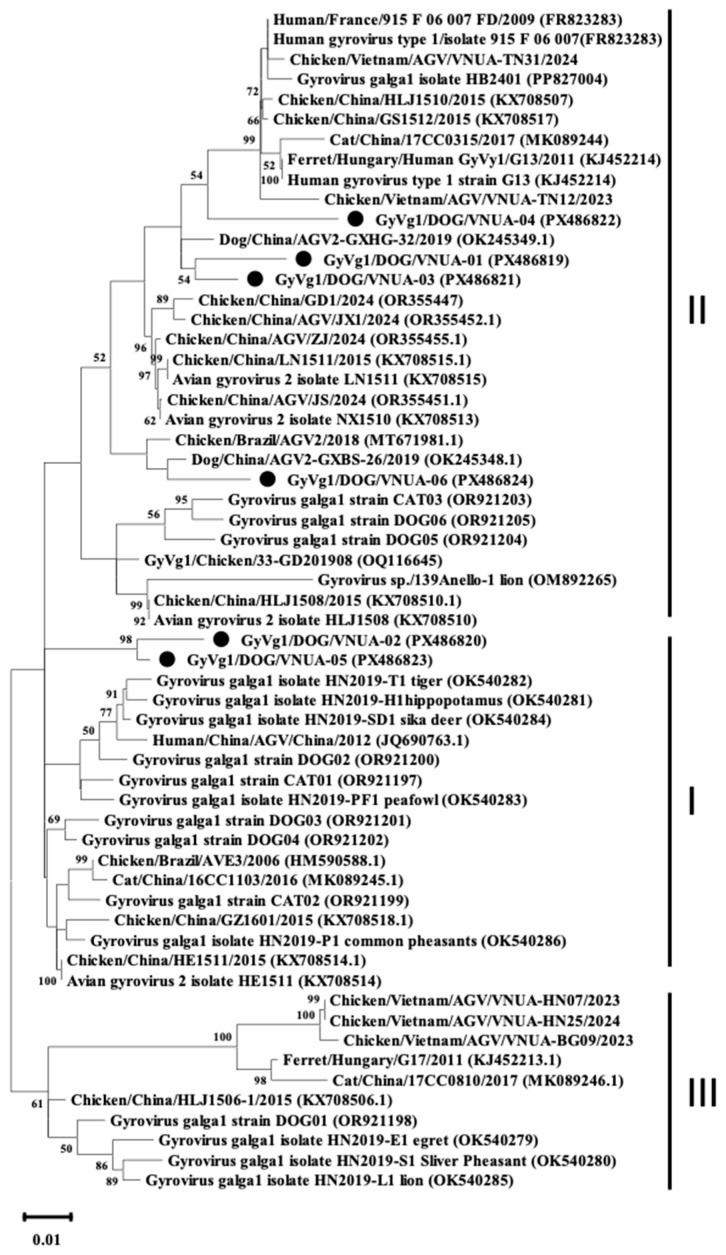
Phylogenetic tree (including 3 clusters: I, II, and III) based on near-complete genome sequences (2375 nt) of current GyVg1 strains from Vietnamese domestic dogs and reference strains available in GenBank was generated using maximum likelihood method in the MEGA X software with 1000 bootstrap replicates. Bootstrap values > 50% are indicated at the nodes. GyVg1 strains identified in the current study are indicated by black circles.

The phylogenetic analysis was performed using the full genome sequences of six representative GyVg1 strains along with other sequences available in the Genbank databases; results indicated that the six GyVg1 strains were formed into two distinct branches, corresponding to GyVg1 cluster I and GyVg1 cluster II (Figure 1). Notably, the current VNUA-02 and VNUA-05 strains clustered together within sub-genotype cluster I (Figure 1). These strains showed a close genetic relationship with the previous Chinese GyVg1 reported in 2022 (Accession no. OR921201 and OR921202). Additionally, the result revealed that the four GyVg1 strains (VNUA-01, VNUA-03, VNUA-04, and VNUA-06) were assigned to genotype cluster II (Figure 1). These strains exhibited close similarities with the Chinese strain reported in 2019 (Accession no. OK245349 and OK245348).

### 2.4. Analysis of Deduced aa Sequence

All six current GyVg1 strains encoded full-length VP1 proteins comprising 460 aa in length. The analysis of the aa sequences of the VP1 proteins showed that no aa changes were found in three motifs at positions 325–329 (FAALS), 363–369 (RRWLTLV), and 412–415 (KAMA). The result reported multiple aa substitutions in the VP1 protein among the analyzed strains. Specifically, aa substitutions were observed at positions 27 (L to Q), 34 (R to P), 36 (G to R), 37 (K to V), and 314 (R to K) (Table 3). Furthermore, VNUA-06 strain exhibited five aa substitutions, including G to S at position 32, R to P at position 34, G to R at position 36, K to V at position 37, and R to K at position 314 (Table 3). Some aa substitutions were detected at positions 34, 35, 36, 41–42, 110–111, and 288–314 across strains VNUA-01, VNUA-02, VNUA-04, and VNUA-05 (Table 3).

The VP2 protein aa sequences of the six current GyVg1 strains identified in the present study consist of 231 aa. Sequence analysis showed that the motif “WLRQCARSHDEICTCGRWRSH” at position 95–115 was conversed among the six GyVg1 strains in this study. There was a novel aa change at position 14 (T/N to Y) of the six GyVg1 VP2 protein. Additionally, aa variations were found at residue 195 (C to S), 202–205 (G202 to R, V204 to R, and N205 to Y), 208 (A to V), and 220–222 (GGS to EDF). Notably, a distinct substitution at 220–222 was identified exclusively in the strain analysis in the present study.

The VP3 aa sequence protein of the six Vietnamese GyVg1 viruses consisted of 125 aa. The VP3 sequences were highly conserved, with only a single aa substitution detected at position 110. This unique substitution was identified exclusively in strain VNUA-06 (Table 4).

### 2.5. Analysis of Selection Pressures Among Current GyVg1 Sequences

Selection profile analysis indicated evidence of positive selection acting on one site in the VP1 protein of all current GyVg1 strains, with a posterior probability of positive selection ≥ 0.9 (Prob[α < β] ≥ 0.9). For the VP2 protein, positive selection was detected at three positions, namely residues 152, 161, and 200 (Table 5). In addition, two positively selected sites, at positions 99 and 115, were observed in the six GyVg1 VP3 proteins in this study.

### 2.6. Recombination Analysis

**Figure 2:**
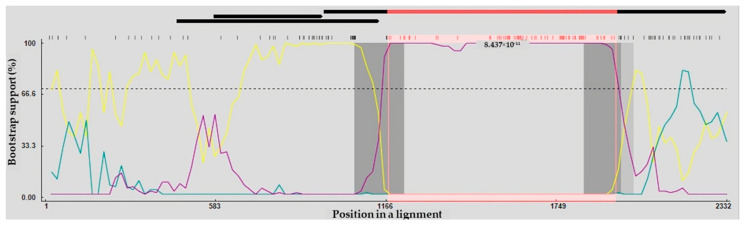
The complete protein-coding regions of Vietnamese GyVg1 viruses were evaluated by BootScan analysis implemented in RDP4, applying the pairwise distance approach, with a window length of 200, a step interval of 20, and 1000 bootstrap iterations. Cat/China/17CC0810/2017– Chicken/China/AGV/JX1/2024 (Major Parent—Minor Parent) (Yellow line). Cat/China/17CC0810/2017—GyVg1/DOG/VNUA-06 (Major Parent—Recombinant) (Blue line). Chicken/China/AGV/JX1/2024—GyVg1/DOG/VNUA-06 (Minor Parent—Recombinant) (Purple line).

Analysis of recombination within the complete VP1 gene sequences indicated that VNUA-06 originated from a recombination event. For strain VNUA-06, Chicken/China/AGV/JX1/2024 and Cat/China/17CC0810/2017 were identified as the putative minor and major parent strains. Six of the nine recombination detection methods in RDP5 provided evidence supporting this potential recombination event (Table 6). The two recombination breakpoints were detected in the VP1 gene sequences of VNUA-06 strain, located at positions corresponding to residues 1174 and 1952 (Figure 2).

## 3. Discussion

In 2008, GyVg1 was initially reported in chickens affected by disease in Brazil. Afterward, GyVg1 has been identified in a broad range of host species, encompassing ferrets, humans, companion animals (dogs and cats), and diverse mammals and avian species in zoo environments. Geographically, GyVg1 viruses have been documented in various parts, including Brazil, Japan, China, South Korea, South Africa, Hungary, and Vietnam [2,7,8,9,15]. Notably, the current study extends the known geographic distribution of GyVg1 by identifying its presence in domestic dogs in Northern Vietnam. It provides the molecular evidence of GyVg1 circulating in companion animals in Vietnam.

GyVg1 genome was identified in 10.71% of fecal samples of domestic dogs obtained from four provinces/cities between 2023 and 2025 in this study. This prevalence exceeds previously reported GyVg1 detection rates of approximately 3.5% in serum dog samples from Central and Eastern China (2020–2023), and 5.66% in the Henan, Shaanxi, and Gansu provinces (2023–2025) [14,19]. However, the samples collected in these studies were not identical, which may have influenced the variability observed in GyVg1 genome prevalence in dogs. Furthermore, the findings support the hypothesis that the prevalence rate may be associated with geographic differences and rearing environments [20]. While the pathogenicity role of GyVg1 remains unclear, GyVg1 has been detected in both diarrheic and asymptomatic dogs. Thus, further studies are necessary to clarify its role in disease.

Regarding GyVg1 in companion animals, GyVg1 was reported in domestic cats in Northeastern China in 2019 and subsequently reported in pet dogs in China in 2022 [12,14]. Evidence from studies conducted from 2020 to 2023 suggested that GyVg1 possesses the potential for cross-species transmission and geographic spread [14]. With ongoing urbanization, companion animals such as pet cats and dogs have become closely integrated into human environments. Their frequent contact with humans raises the capacity that these animals may function as reservoirs or intermediate hosts for emerging viruses, including GyVg1. These findings warrant further investigation into possible public health risks.

In the study, the complete genomes of six GyVg1 strains were 2375 nt in length, notably shorter than those of the approximately 2380 nt GyVg1 genomes reported in GenBank. This difference may be explained by variations in the lengths of the poly-C and poly-G regions upstream of the 5′-URT [21]. Additionally, the six GyVg1 complete genome exhibited over 95% nt identity with other GyVg1 available in GenBank. This finding is in agreement with the previous observations, suggesting that GyVg1 strains currently circulating in Vietnam remain highly conserved at the genomic level [17,22].

According to the full-genome phylogenetic analysis, the GyVg1 viruses detected in domestic dogs were clustered separately from reference strains obtained from other hosts or geographic regions. The presence of both similarity and differential clustering of strains in the phylogenetic tree implies that the evolutionary complexity of GyVg1 may result from its wide host spectrum, indicating a potential for GyVg1 recombination. Accumulating evidence from the previous studies has provided the occurrence of genetic recombination in Gyrovirus [13,22,23,24]. In the current study, evidence of recombination was identified in the Vietnamese GyVg1 viruses within the VP1 protein region. The recombination analysis suggests that GyVg/DOG/VNUA-06 resulted from a combination of a major parental strain, Cat/China/17CC0810/2017 (identified in cat, China, 2017), and a minor parental strain, Chicken/China/JX1/2024 (identified in chicken, China, 2024). The result further implies that GyVg1 may undergo complex horizontal transmission pathways. In addition, this finding is strongly consistent with prior reports showing that the GyVg1 genome contains multiple recombination regions, such as the VP1 coding, non-coding, and overlapped regions [22].

As a major capsid protein of GyVg1, the VP1 protein is involved in receptor binding and virus–host interaction [21]. According to previous reports, VP1 contains a hypervariable region spanning aa residues 288 to 314 [17,23]. In the present study, multiple aa substitutions at positions 288 and 314 were observed within this hypervariable region of all six current GyVg1 strains. Moreover, several aa substitutions were identified in other regions of the VP1 protein. Nevertheless, further investigations are necessary to evaluate whether these substitutions affect GyVg1 host adaptation.

Similar to CAV, one member of Gyrovirus, the VP2 protein of GyVg1 contains a conserved ‘WLRQCARSHDEICTCGRWRSH’ motif (residues 95–115), which was identified in all six Vietnamese GyVg1 strains. This motif has been reported to be involved in VP2 site-directed mutagenesis. It is thought to play an important role in impairing viral particle replication. Additionally, the VP2 protein of the obtained strains contained multiple representative aa mutations, some of which were unique to these strains. The GyVg1 VP3 protein is known to induce apoptosis of tumor cells, with several functional domains predicted to contribute to this activity [24,25,26]. In the present study, there was only aa substitution at the residues 110 found in the VP3 protein. Additional experimental investigations are necessary to determine whether these mutations affect the molecular functions of GyVg1 VP2 and VP3.

## 4. Materials and Methods

### 4.1. Ethics Statement

All procedures involving animals (including handling and sample collection) were carried out in rigorous compliance with the Vietnam National University of Agriculture’s ethical regulations (CARE-2023/08 approval at 25 March 2023). The study protocol was reviewed and approved by the University’s Committee on Animal Research and Ethics. Prior to sampling, written informed consent was obtained from all dog owners after a clear explanation of study objectives and procedures. Sample collection was performed in a manner that minimized animal stress and discomfort, in accordance with internationally recognized animal welfare standards.

### 4.2. Samples

From April 2023 to September 2025, a total of 168 fecal samples were obtained from domestic dogs in four provinces/cities in Northern Vietnam, namely Hanoi (HN), Haiphong (HP), Bacninh (BN), and Hungyen (HY), including both diarrheic and clinically healthy dogs. Notably, diarrheic samples were obtained only from BN and HY. Each sample was processed by preparing a 10% (*w*/*v*) tissue homogenate in phosphate-buffered saline containing gentamicin (10 mg/mL) and preserved at −80 °C at the laboratory at Faculty of Veterinary Medicine, Vietnam National University of Agriculture, Vietnam until further analysis.

### 4.3. DNA Extraction and PCR for GyVg1 Genome Detection

DNA extraction was performed on homogenized samples using the Viral Gene-spin™ Viral DNA/RNA Extraction Kit (iNtRON Biotechnology, Seoul, Republic of Korea), in accordance with the manufacturer’s recommended protocol.

For GyVg1 detection, GyVg1/F and GyVg1/R primers were employed to amplify the target of 344 bp, a genomic region corresponding to partial VP2 and VP3 genes (Table 7), as previously described. Sequencing was demonstrated using three overlapping primer sets that generated amplicons of 802 or 801 bp, 733 bp, and 981 bp, spanning the GyVg1 genome (Table 7). PCR protocol included an initial denaturation at 95 °C for 5 min, 40 cycles of amplification (95 °C for 30 s, 60 °C for 30 s, and 72 °C for 40 s), and a final extension step at 72 °C for 10 min. The PCR amplicons were separated by 1.2% agarose gel electrophoresis and visualized by UV illumination.

### 4.4. Nucleotide Sequencing and Phylogenetic Analysis

The PCR amplicons were purified using GeneClean^®^ II Kits (MP Biomedicals, Santa Ana, CA, USA), followed by sequencing of the GyVg1 VP1–VP3 genes by 1st BASE, Malaysia.

Sequence analysis was performed using GENETYX version 10 software (GENETYX Corp., Tokyo, Japan) and compared with available reference sequences through BLAST+ 2.17.0 homology searches (https://blast.ncbi.nlm.nih.gov/Blast.cgi, accessed on 16 September 2025). Amino acid (aa) sequences deduced were aligned using the Clustal W algorithm of the BioEdit (version 7.2) [27,28]. Evolutionary distances were estimated from the aligned sequences using the Kimura 2-parameter model. Phylogenetic trees were constructed using the Maximum Likelihood (ML) method with 1000 bootstrap replicates in MEGA X software [29].

### 4.5. Analysis of Recombination Events, Evolutionary Distances, and Selection Profiles

Putative recombination events were identified using the Recombination Detection Program (RDP), version Beta 4.97 [18]. Evolution distances were estimated using the MEGA X software based on the Maximum Composite Likelihood model [29,30]. Analyses of evolutionary selection profiles were conducted using Datamonkey (http://www.datamonkey.org/, accessed on 25 November 2025), according to the Fast Unconstrained Bayesian AppRoximation (FUBAR) method [31].

### 4.6. Accession Numbers of Nucleotide Sequence

The nucleotide sequences of GyVg1 viruses have been deposited in the GenBank database under accession numbers PX486819–PX486824 (http://www.ncbi.nlm.nih.gov/Genbank/, accessed at 22 March 2027).

### 4.7. Data Analysis and Statistics

Fisher’s exact test was applied to evaluate significant differences in GyVg1 genome detection rates among geographical regions. Statistical significance was defined as *p*-value < 0.05.

## 5. Conclusions

In summary, this current study reports the prevalence of GyVg1 in domestic dogs in Vietnam during 2023–2025 and presents detailed genetic characteristics of the GyVg1 strain circulating in Northern Vietnam. These findings contribute valuable data for further studies on GyVg1 evolution and emphasize the importance of molecular characterization in understanding GyVg1 diversity.

## Figures and Tables

**Table 1:** Detection of the GyVg1 genome in dogs in different regions of Northern Vietnam.

Province/City	No. of Sample Collection	No. of Positive Samples (%)	95% CI
Hanoi (HN)	82	8 (9.75)	4.31–18.32
Haiphong (HP)	26	3 (11.53)	2.45–30.15
Bacninh (BN)	45	5 (11.11)	3.71–24.05
Hungyen (HY)	15	2 (13.33)	1.66–40.46
Total	168	18 (10.71)	6.48–16.40

**Table 2:** Nucleotide sequence similarities between the full genome of the obtained GyVg1 strains detected in domestic dogs and those of representative reference strains.

Cluster	Strain Name	No. of Strains/nt Similarity (%)										
		1	2	3	4	5	6	7	8	9	10	11
I	1. DOG02 (OR921200) ^a^											
I	2. DOG04 (OR921202) ^a^	98.02										
II	3. DOG/AGV2-GXHG-32/2019 (OK245349) ^a^	96.13	97.16									
II	4. DOG/AGV2-GXBS-26/2019 (OK245348) ^a^	96.60	97.63	98.92								
III	5. CAT/17CC0810/2017 (MK089246) ^a^	95.52	95.09	95.39	95.01							
III	6. DOG01 (OR921198) ^a^	97.25	97.85	96.77	97.24	96.04						
	7. VNUA-01 (PX486819) ^a^	95.19	96.96	97.59	97.03	94.36	95.58					
	8. VNUA-02 (PX486820) ^a^	96.35	97.64	96.77	96.77	95.27	96.09	**96.05**				
	9. VNUA-03 (PX486821) ^a^	95.84	96.65	97.80	97.80	95.18	96.39	**97.85**	**96.48**			
	10. VNUA-04 (PX486822) ^a^	95.15	95.79	96.60	96.60	94.62	95.24	**96.09**	**96.09**	**97.21**		
	11. VNUA-05 (PX486823) ^a^	97.64	97.89	96.51	96.51	94.84	96.61	**94.98**	**98.62**	**95.54**	**95.15**	
	12. VNUA-06 (PX486824) ^a^	96.09	97.08	98.32	98.32	94.41	96.82	**96.82**	**95.88**	**98.54**	**96.44**	**95.79**

The highest full genome identity between Vietnamese GyVg1 strains and representative reference strains is underlined, while nucleotide identities among the full genome of the Vietnamese GyVg1 strains are shown in bold. ^a^ GenBank accession number. Details of the reference strains evaluated in Table 2 are presented in Supplemental Table S3.

**Table 3:** Variations in the VP1 aa sequences of the obtained GyVg1 viruses compared with other available GyVg1 viruses in the GenBank database.

GyVg1 Strains	VP1 Protein								
	27	32	34	35	36	37	41–42	110–111	288–314
GyVg1 * (I–III)	L	Y	R	R	G	K	AR	NL	V^288^ … EI^310–311^ … R^314^
VNUA-01	. ^a^	.	.	.	.	.	GP	.	… K^314^
VNUA-02	.	.	.	.	.	.	GP	.	M^288^ … KN^310–311^ …
VNUA-03	Q	.	P	.	R	V	.	.	… K^314^
VNUA-04	.	.	G	E	K	.	.	TF	L^288^
VNUA-05	.	.	.	.	.	.	.	.	M^288^ … KN^310–311^ …
VNUA-06	.	F	P	.	R	V	.	.	… K^314^

* The available GyVg1 group into cluster I–III from GenBank. ^a^ same as the available GyVg1 sequence.

**Table 4:** Variations in the VP2 and VP3 aa sequences of the Vietnamese GyVg1 viruses compared with other available GyVg1 viruses in the GenBank database.

GyVg1 Strains	VP2 Protein									VP3 Protein
	14	95–115	140	156–158	195	202–205	208	213	220–222	110
GyVg1 * (I–III)	T/N	WLRQCARSHDEICTCGRWRSH	Q/R	GKR	C	GGVN	A	P	GGS	I
VNUA-01	Y	.	.	.	.	.	.	.	.	.
VNUA-02	Y	.	H	.	.	G^204^	.	.	.	.
VNUA-03	Y	.	.	.	S	R^202^.R^204^Y^205^	V	.	EDF	.
VNUA-04	Y	.	.	.	.	R^203^	.	.	.	.
VNUA-05	Y	.	.	RRG	.	.	.	H	.	.
VNUA-06	Y	.	.	RRG	.	R^202^.G^204^Y^205^	.	.	.	V

* The representative GyVg1 belong to clusters I–III from GenBank.

**Table 5:** Positive selection in VP (1–3) sequences of the current GyVg1 viruses from Vietnamese domestic dogs.

Protein	aa Position	α	β	β–α	Prob[α > β]	Prob[α < β]	BayesIndex[α < β]
VP1	34	0.97	12.13	11.16	0.07	0.90	14.54
VP2	152	1.85	12.35	10.50	0.07	0.90	11.23
	161	1.90	12.90	11.00	0.07	0.90	11.32
	200	1.52	13.40	11.88	0.05	0.92	13.98
VP3	99	2.52	22.83	20.31	0.04	0.93	14.89
	115	2.37	19.41	17.03	0.05	0.93	13.86

α represents posterior synonymous substitution rate at a site, whereas β represents posterior non-synonymous substitution rate at a site; negative selection when α > β; positive selection when α < β; neutral selection when α = β. A posterior probability of negative selection is defined as Prob[α > β] ≥ 0.9, while posterior probability of positive selection is defined as Prob[α < β] ≥ 0.9.

**Table 6:** Recombination statistics of GyVg1/DOG/VNUA-06 using RDP5.

No.	Method	Recombination *p*-Value
1	RPD	3.55 × 10^−4^
2	GENECONV	1.91 × 10^−8^
3	BootScan	5.85 × 10^−5^
4	MaxChi	9.89 × 10^−8^
5	SiScan	1.71 × 10^−14^
6	3Seq	1.87 × 10^−7^

*p*-value < 0.05: Recombination events occurred.

**Table 7:** The information on primers used in this study.

Primer’s Name	Sequence (5′–3′) of Primer	Amplicon Length (bp)	References
GyVg/F1	CGTGTCCGCCAGCAGAAAC	344	[14]
GyVg/R1	GGTAGAAGCCAAAGCGTCCAC		
GyVg/QC1F	ATTTCCTAGCAC TCAAAAACCCATT	802	[21]
GyVg/QC1R	TCTGGGCGTGCTCAATTCTGATT		
GyVg/QC2F	TCACAGCCAATCAGAATTGAGCACG	733	
GyVg/QC2R	TTCTACGCGCATATCGAAATTTACC		
GyVg/QC3F	TATTCCCGGAGGGGTAAATTTCGAT	981	
GyVg/QC3R	CCCCTGTCCCCGTGATGGAATGTTT		

## Data Availability

The original data supporting the findings of this study are provided in the article/Supplementary Materials. Further inquiries can be addressed to the corresponding author.

## Supplementary Materials

The following supporting information can be downloaded at: https://www.mdpi.com/article/10.3390/ijms27031384/s1.
