# Supplementary material for: Molecular Characterization of Gyrovirus galga 1 in Domestic Dogs in the North of Vietnam Indicates the Presence of Recombination Events
Source: Int J Mol Sci. 2026 Jan 30;27(3):1384. doi: 10.3390/ijms27031384 (PMC12898029; doi:10.3390/ijms27031384)
Supplement: Supplementary file 1 [file ijms-27-01384-s001.zip › ijms-4095651-supplementary.pdf]

Supplemental Table 1. Features of GyVg1 isolates obtained in this study

Virus strains	Year	District	Status	GenBank accession no.
VNUA-01	2025	Hanoi	Healthy	PX486819
VNUA-02	2025	Hanoi	Healthy	PX486820
VNUA-03	2023	Hungyen	Diarrheic	PX486821
VNUA-04	2024	Haiphong	Healthy	PX486822
VNUA-05	2023	Bacninh	Diarrheic	PX486823
VNUA-06	2025	Bacninh	Diarrheic	PX486824

Supplemental Table 2. Nucleotide sequence similarities between the full genome of Vietnamese GyVg1 strains detected in domestic dogs and those of Vietnamese chicken GyVg1 strains

Strain name	No. of strains/nt identity (%)					
	VNUA_01	VNUA_02	VNUA_03	VNUA_04	VNUA_05	VNUA_06
VNUA-TN12/2023 (PQ154631)[a]	96.04	97.29	96.81	96.34	96.43	95.87
VNUA-HN07/2023 (PQ154632)	93.63	95.18	94.49	93.93	95.57	94.53
VNUA-TN31/2024 (PQ154633)	96.06	96.99	97.37	96.90	96.04	96.43
VNUA-BG09/2023 (PQ154634)	93.59	95.14	94.36	93.76	95.57	94.36
VNUA-HN25/2024 (PQ154635)	93.63	95.18	94.49	93.93	95.57	94.53

[a] GenBank accession number.

Supplemental Table 3. Description of GyVg1 strains used in this study.

Virus strains	Year	Location	Source	GenBank accession no.
DOG02	2021	China	Serum, dog	OR921200
DOG04	2022	China	Serum, dog	OR921202
DOG/AGV2-GXHG-32/2019	2019	China	Serum, canine	OK245349
DOG/AGV2-GXBS-26/2019	2019	China	Serum, canine	OK245348
CAT/17CC0810/2017	2017	China	Feces, cat	MK089246
DOG01	2021	China	Serum, dog	OR921198
